# Small Hydrophobic Protein of Human Metapneumovirus Does Not Affect Virus Replication and Host Gene Expression *In Vitro*

**DOI:** 10.1371/journal.pone.0058572

**Published:** 2013-03-06

**Authors:** Miranda de Graaf, Sander Herfst, Jamil Aarbiou, Peter C. Burgers, Fatiha Zaaraoui-Boutahar, Maarten Bijl, Wilfred van IJcken, Eefje J. A. Schrauwen, Albert D. M. E. Osterhaus, Theo M. Luider, Bob J. Scholte, Ron A. M. Fouchier, Arno C. Andeweg

**Affiliations:** 1 Department of Viroscience, Erasmus Medical Center, Rotterdam, The Netherlands; 2 Department of Cell Biology, Erasmus Medical Center, Rotterdam, The Netherlands; 3 Department of Neurology, Erasmus Medical Center, Rotterdam, The Netherlands; 4 Center for Biomics, Erasmus Medical Center, Rotterdam, The Netherlands; 5 Department of Zoology, University of Cambridge, Cambridge, United Kingdom; College of Medicine, Hallym University, Korea, Republic Of

## Abstract

Human metapneumovirus (HMPV) encodes a small hydrophobic (SH) protein of unknown function. HMPV from which the SH open reading frame was deleted (HMPVΔSH) was viable and displayed similar replication kinetics, cytopathic effect and plaque size compared with wild type HMPV in several cell-lines. In addition, no differences were observed in infection efficiency or cell-to-cell spreading in human primary bronchial epithelial cells (HPBEC) cultured at an air-liquid interphase. Host gene expression was analyzed in A549 cells infected with HMPV or HMPVΔSH using microarrays and mass spectrometry (MS) based techniques at multiple time points post infection. Only minor differences were observed in mRNA or protein expression levels. A possible function of HMPV SH as apoptosis blocker, as proposed for several members of the family *Paramyxoviridae*, was rejected based on this analysis. So far, a clear phenotype of HMPV SH deletion mutants *in vitro* at the virus and host levels is absent.

## Introduction

Since its discovery in 2001, the epidemiology, prevalence, and clinical signs of human metapneumovirus (HMPV) have been studied extensively [1], [2], [3], [4], [5]. Based on genetic and antigenic analyses, four sublineages of HMPV (A1, A2, B1 and B2) have been identified [1], [6]. Reverse genetics systems are now available for all four sublineages facilitating fundamental and applied research [7], [8].

The non-segmented negative sense genome of HMPV encodes at least 9 putative open reading frames (ORFs); from the 3’ to 5’ ends: nucleoprotein (N), phosphoprotein (P), matrix protein (M), fusion protein (F), M2-1 and M2-2, small hydrophobic protein (SH), attachment protein (G), and large polymerase protein (L) [9]. For most of these ORFs a possible function has been assigned based on homologies of closely related viruses such as the human respiratory syncytial virus (HRSV). However, several studies have demonstrated that there are functional differences between the ORFs of HRSV and HMPV. For example, HRSV M2.1 was described as a transcriptional elongation factor that is required for virus viability [10], while recombinant HMPV can be recovered in the absence of M2.1 Furthermore HMPV M2.1 deletion mutants replicated efficiently *in vitro* but not *in vivo* [7], [11]. Similarly, in contrast to the HRSV G protein, the HMPV G protein is not essential for replication *in vivo* [12], [13].

The enveloped HMPV particles potentially contain three virus encoded transmembrane glycoproteins, the F, G and SH proteins. A function for the SH protein cannot readably be assigned for HMPV since its function is largely unclear for other members of the genus *Pneumovirinae*. The SH protein of mumps virus (MuV) is a type I transmembrane protein, whereas the SH proteins of other paramyxoviruses are type II transmembrane proteins. The HMPV SH protein was found to be expressed in several different forms in virus infected cells depending on the glycosylation status: an unglycosylated form (SH0), an N-glycosylated form (SHg1) and a more extensively glycosylated form (SHg2) [12], [14]. The SH protein of HMPV is the largest among the members of the family *Paramyxoviridae*, 183 aa for HMPV-A and 177 aa for HMPV-B compared to avian metapneumovirus subgroup A (AMPV-A) (81 aa), AMPV-B (175 aa), AMPV-C (177 aa), HRSV (64 aa), bovine respiratory syncytial virus (BRSV) (81 aa), MuV (57 aa) and parainfluenza virus type 5 (PIV5) (44 aa). Sequence similarity between HMPV and AMPV-C was found to be low and there is no discernable sequence identity with the SH proteins of other viruses [9], [15].

HRSV from which SH was deleted was previously found to replicate efficiently in cell culture and slightly less efficient in the higher upper respiratory tract of mice and in the lower respiratory tract of chimpanzees [16], [17]. In contrast, HMPV SH deletion mutants replicated with an efficiency similar to that of wild type HMPV in the upper and lower respiratory tract of hamsters [12] and replicated only marginally less efficient in non-human primates [18]. The SH proteins of members of the family *Paramyxoviridae* have been suggested to act as a viroporin [19], [20], or to have a function in blocking the TNF-α-mediated apoptosis pathway [14], [21], [22], [23]. PIV5 from which the SH was deleted (PIV5ΔSH) was viable and displayed similar replication kinetics and plaque size compared to the wild type virus, but caused increased cytopathic effect (CPE) in MDBK and L929 cells, via TNF-α-mediated apoptosis [23], [24].

To study the function of the SH protein of HMPV, SH deletion mutants were generated using a wild type HMPV or HMPV encoding green fluorescent protein (GFP) as backbone [25]. These deletion mutants replicated with similar efficiency as the parental viruses in Vero-118 cells and human primary bronchial epithelial cells (HPBEC) cultured at air-liquid interphase. Only minor differences were observed in host gene or protein expression levels using microarrays and mass spectrometry (MS) based methods upon infection of the A549 lung fibroblast cell line with HMPV or HMPV SH deletion mutants. Based on this study it was concluded that the SH protein of HMPV has no identifiable function in the context of the virus and host cells *in vitro*.

## Results

### Replication characteristics of HMPV SH deletion mutants in tissue culture

**Figure 1:**
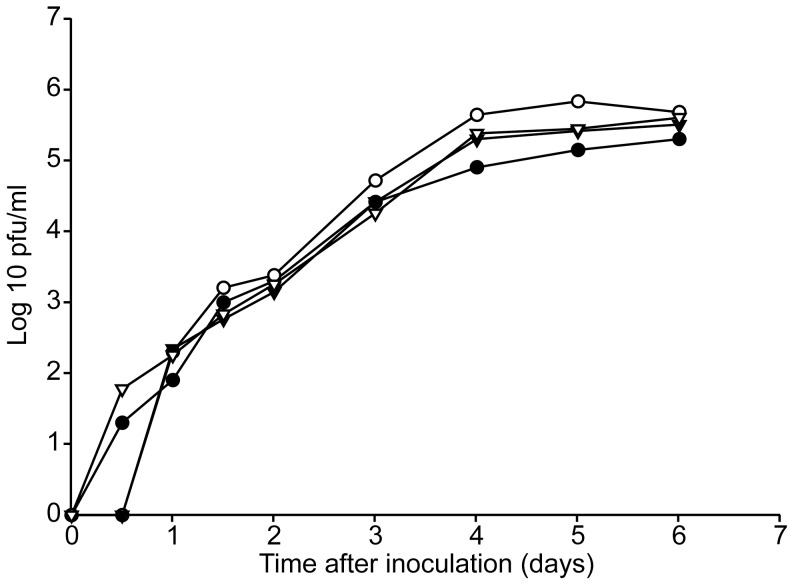
Replication kinetics of HMPV and SH deletion mutants. Vero-118 cells were inoculated at a MOI of 0.1 with HMPV (closed circle), HMPVΔSH (open circle)_,_ HMPV-GFP (closed triangle down) and HMPVΔSH-GFP (open triangle down). Supernatants were collected daily and virus titres were determined by plaque assay.

**Figure 2:**
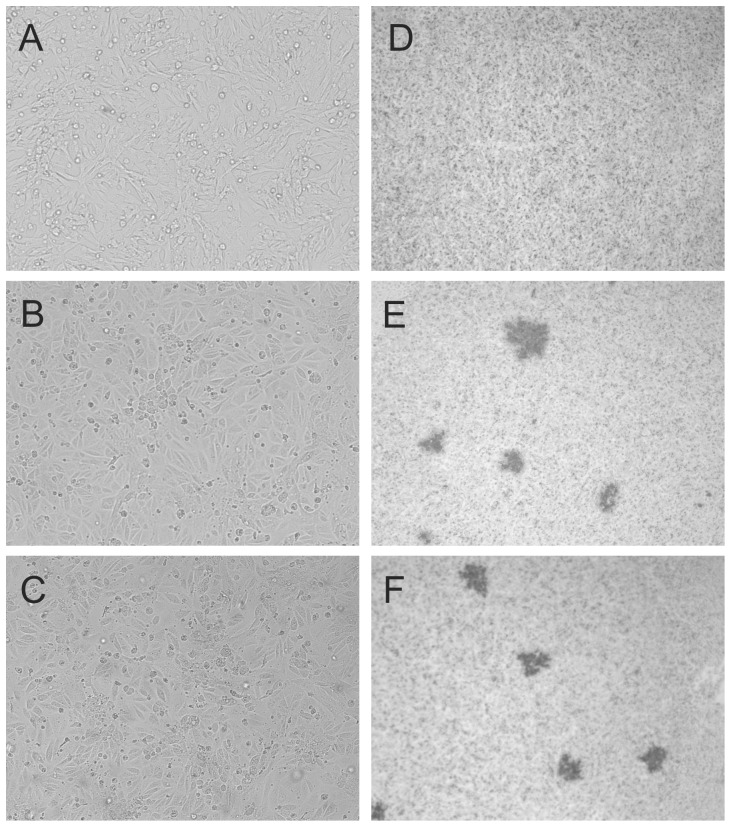
Cytopathic effect (CPE, left panels) or plaques (right panels) in mock (a and d), wild type HMPV (b and e) or HMPVΔSH (c and f) inoculated Vero-118 cells. Left panels, Vero-118 cells were inoculated at a MOI of 0.1 and were subsequently photographed without further treatment 6 days after inoculation. Right panels, Vero-118 cells were inoculated and incubated with a methylcellulose overlay. Plaques were visualized by immunostaining 6 days after inoculation.

To investigate the function of the HMPV SH protein, SH deletion mutants were generated by removing the SH ORF, resulting in HMPVΔSH. A similar deletion mutant was generated using a recombinant HMPV expressing GFP as backbone virus, resulting in HMPVΔSH-GFP. Replication curves were generated to compare the replication kinetics of HMPV SH deletion mutants with those of parental HMPV (Figure 1). There were no apparent differences in replication kinetics between wild type HMPV and HMPVΔSH confirming that the SH protein is dispensable *in-vitro* in Vero-118 cells. Moreover, HMPV-GFP and HMPVΔSH-GFP replicated to similar titers as wild type HMPV as well. For RSV, the deletion of SH did not alter the replication kinetics or production of syncytia but did result in plaques which were 70% larger than plaques produced by wild type RSV [17]. To investigate the impact of the HMPV SH deletion on CPE, Vero-118 cells infected with HMPV and HMPVΔSH were photographed five days after inoculation (Figure 2, left panels). CPE was indistinguishable between HMPV and HMPVΔSH infected Vero-118 cells; both viruses yielded focal rounding and detachment of cells and no syncytium formation. Plaque assays performed with Vero-118 cells inoculated with HMPV or HMPVΔSH and overlaid with methylcellulose revealed that plaque sizes were similar (Figure 2, right panels).

### Analysis of SH expression

**Figure 3:**
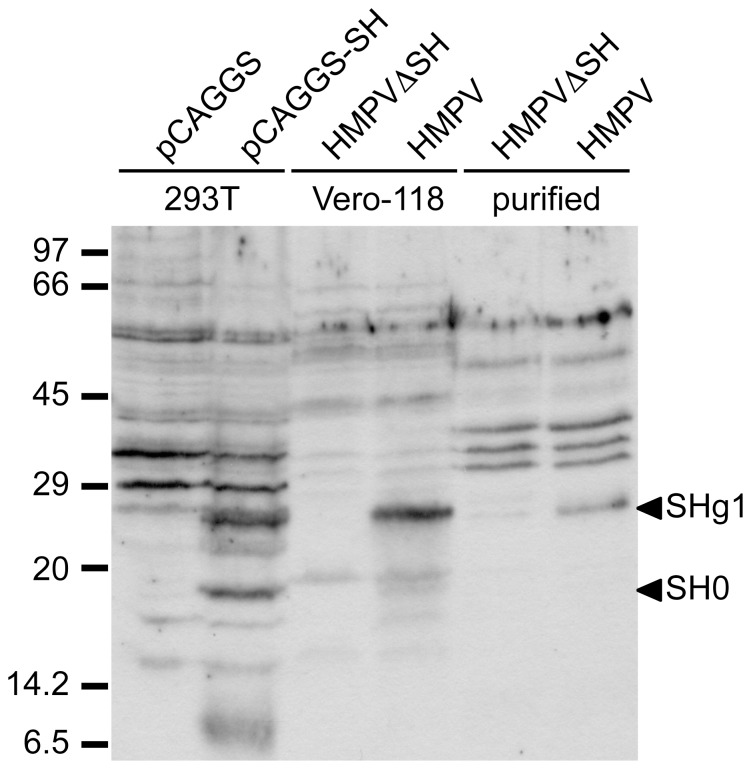
Western blot analysis of the SH protein. 293T cells transfected with pCAGGS (lane 1), pCAGGS-SH (lane 2), Vero-118 cells infected with HMPV (lane 3) or HMPV ΔSH (lanes 4) and purified virions of HMPVΔSH (lane 5) and HMPV (lane 6) were analyzed on 12.5% polyacryamide gels. The SH protein was detected using rabbit serum raised against a mixture of petides representing aa 2–16 and 95–110 of the SH protein.

Expression of the SH protein in HMPV-infected cells and virions was analyzed by Western blot (Figure 3). Vero-118 cells were inoculated with HMPV or HMPVΔSH and cells and the supernatant were harvested 7 days post inoculation (p.i.). Virus-particle-containing supernatant was subsequently concentrated and purified on sucrose gradients. 293T cells transfected with a plasmid expressing the SH protein (pCAGGS-SH) served as a positive control for SH expression. Two additional bands were observed for samples containing the SH protein: 293T cells transfected with pCAGGS-SH (lane 2), cells infected with HMPV (lane 4) and purified HMPV virions (lane 6). These bands of 19 and 26 kDa were not detected for 293T cells transfected with PCAGGS (lane 1), cell infected with HMPVΔSH (lane 3) and purified HMPVΔSH virions (lane 5). The band of 19 kDa corresponds to the calculated size (20.1 kDa) of the non-glycosylated form of the SH protein and is designated SH0. The other band of 26 kDa (designated SHg1) presumably represents the N-glycosylated form [12].

In addition to Western blot analysis, the differential expression of the SH protein for HMPV and HMPVΔSH virions was confirmed by nano Liquid Chromatography (LC) Electrospray Ionization (ESI) Orbitrap Mass Spectrometry (MS) analysis. To specifically detect the SH protein, samples were run employing an inclusion list of 13 theoretical tryptic SH peptides (Table S1). The peptide VENNLQACQPK of the SH protein was found for the purified HMPV but not for the purified HMPVΔSH virions. Unlike most of the theoretical tryptic SH peptides, VENNLQACQPK does not contain potential glycosylation sites. The peptide was found with a Mascot ion score of 42 with a significant threshold of p<0.01 and zero modifications, confirming the expression of the SH protein.

### Replication characteristics of HMPV and HMPVΔSH in HPBEC

**Figure 4:**
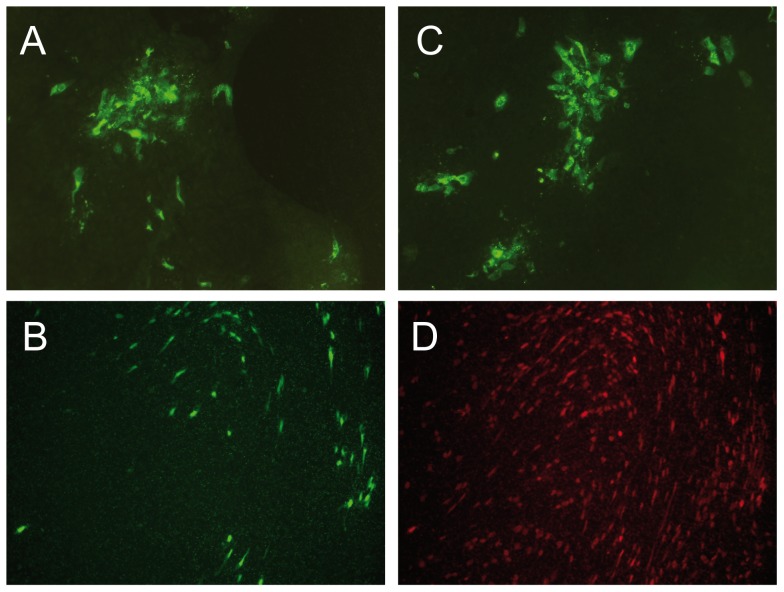
HPBEC cultured at air-liquid interphase were inoculated with wild type HMPV (a, b and d) or HMPVΔSH (c) at a MOI of 4. Six days after inoculation, infected cells were visualized by immunostaining with HMPV specific polyclonal anti-serum (a and c). The HMPV-infected cells from panel b and d are the same field of cells, double stained for HMPV infected cells (b) and ciliated cells by staining with anti ß-tubulin antibodies (d).

HMPV is known to replicate primarily in ciliated respiratory epithelial cells in macaques [26]. Therefore we next investigated the phenotype of HMPV and HMPVΔSH in air-liquid interphase cultures of HPBEC, as these cultures have a pseudo stratified epithelial phenotype and consist of both ciliated and mucus producing cells, similar to airway epithelium *in vivo* [27]. Cells were pre-treated with lysophosphatidylcholine (LPC) to enhance infection efficiency, and inoculated with HMPV or HMPVΔSH. Every day, fresh medium containing trypsin was added and mucus overlays were washed. HMPV and HMPVΔSH both replicated in HPBEC but no differences were observed in virus spread or the number of virus infected cells between the two viruses (Figure 4a and c). A separate staining specific for ciliated cells was performed, revealing that primarily ciliated cells were infected with HMPV (Figure 4b and d).

### MRNA profiling of HMPV- and HMPVΔSH-infected A549 cells

**Figure 5:**
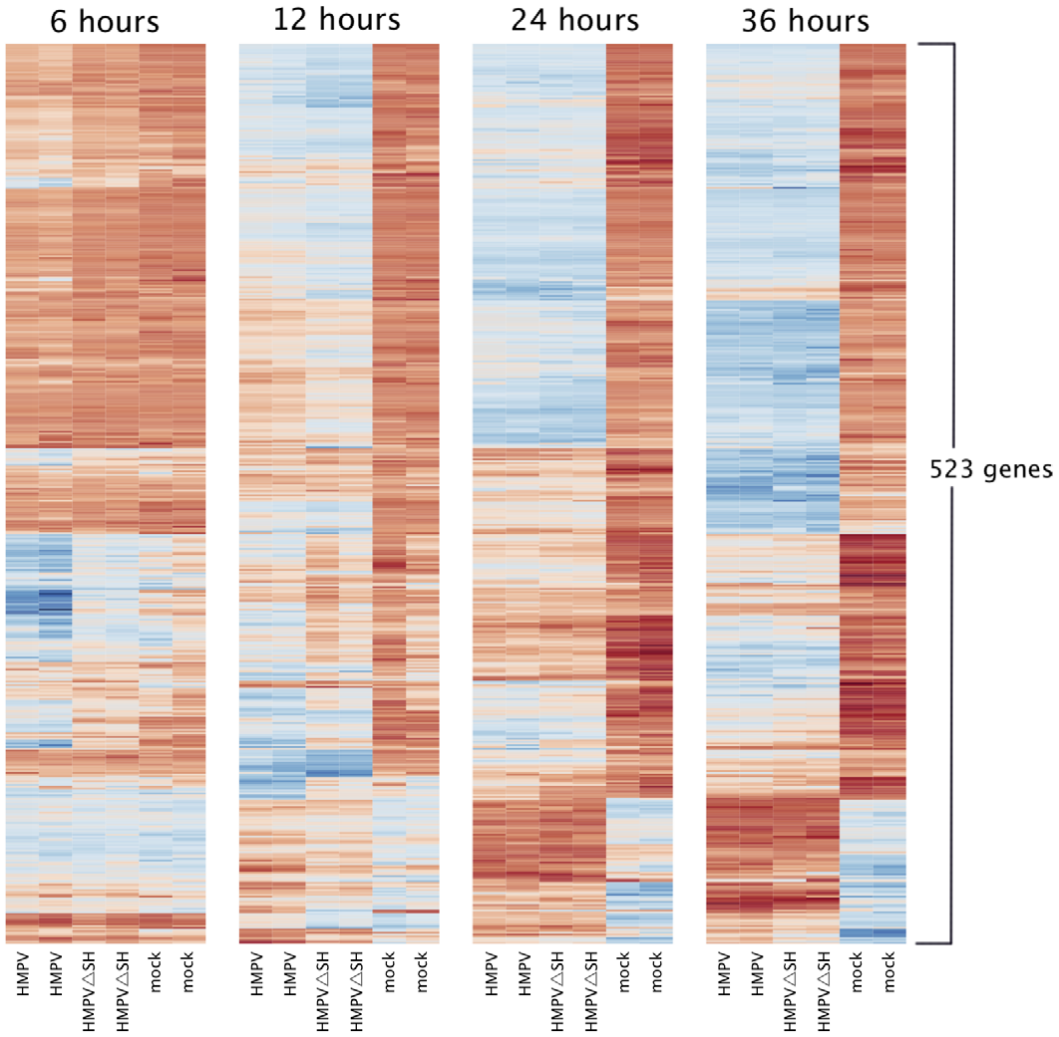
Heat map displaying differential expression of genes of A549 cells following inoculation with wild type HMPV, HMPVΔSH or mock 6, 12, 24, or 36 hours in duplicate (Induced blue, repressed red values).

Messenger RNA profiling was performed to identify and characterize differences in the host response to HMPV and HMPVΔSH infection. To this end, A549 cells inoculated with HMPV, HMPVΔSH or medium were harvested 6, 12, 24 and 36 hours p.i. for microarray analysis using Affymetrix HG-U133 plus 2.0 GeneChips. Differentially expressed host genes between HMPV, HMPVΔSH and mock infected cells were identified using limma analysis. The analysis revealed that 523 out of the 19,006 genes that were studied were differentially expressed in at least one of the three pairwise comparisons, at any time point, when applying a false discovery rate (FDR) of smaller or equal to 0.05 and a 2log fold change threshold of 1 (equals an absolute fold change of 2). Increasing over time, HMPV and HMPVΔSH infections both affected the gene expression level of more than 300 genes (Table 1). Pathway analysis revealed that the function of these differentially expressed genes was most significantly associated with (innate) biological processes like apoptosis, immune cell trafficking, cell-to-cell signaling and cell-cell interactions. The results were remarkably similar to results found for wild type HMPV infection by Bao *et al* (30). The fold change of these up- or down regulated genes, relative to base line expression levels as measured in mock infected cells, ranged up to 7 (equals an absolute fold change of 128, Table 2 and 3). Figure 5 shows that HMPV and HMPVΔSH infections change the expression level of a similar set of genes in a very similar fashion. Limma analysis of the genome wide expression profiles indeed revealed that only 35 genes were significantly differentially regulated when comparing the HMPV and HMPVΔSH infections directly (Table 1, 2 and 3). Pathway analysis revealed that these genes were associated with extravasation, recruitment, adhesion and infiltration of cells of lymphoid origin, apoptosis, cell activation and differentiation, and interferon signaling.

**Table 1:** Number of up- and down-regulated genes in pairwise comparisons following inoculation of A549 cells with HMPV, HMPVΔSH or mock inoculated A549 cells at 6, 12, 24 and 36 hours.

	Up-regluated*			Down-regulated*		
hours p.i.	HMPV vs HMPVΔSH	HMPV vs mock	HMPVΔSH vs mock	HMPV vs HMPVΔSH	HMPV vs mock	HMPVΔSH vs mock
6	20	73	14	0	0	0
12	5	185	215	12	5	1
24	0	195	188	0	16	15
36	1	328	315	1	61	51

* Applying a 2log fold change of 1 and a FDR < =  0.05.

**Table 2:** Up-regulated genes (FDR < =  0.05) with a minimum fold change of 1 (2log values) in A549 cells infected with HMPV compared with HMPVΔSH infected cells (column 3).

Symbol	Gene name	HMPV vs HMPVΔSH	HMPV vs mock	HMPVΔSH vs mock	hours p.i.
TCHH	trichohyalin	1.59	2.07	n.s.*	6
CXCL1	chemokine (C-X-C motif) ligand 1 (melanoma growth stimulating activity, alpha)	1.56	3.12	1.56	6
SERPINA3	serpin peptidase inhibitor, clade A (alpha-1 antiproteinase, antitrypsin), member 3	1.45	2.64	1.20	6
STAT5A	signal transducer and activator of transcription 5A	1.35	1.78	n.s.	6
ARL14	ADP-ribosylation factor-like 14	1.33	1.33	n.s.	6
MMP10	matrix metallopeptidase 10 (stromelysin 2)	1.33	1.46	n.s.	6
MX1	myxovirus (influenza virus) resistance 1, interferon-inducible protein p78 (mouse)	1.25	2.64	1.39	6
GBP1	guanylate binding protein 1, interferon-inducible, 67kDa	1.23	2.13	0.90	6
CCL5	chemokine (C-C motif) ligand 5	1.23	2.01	0.78	6
LAMB3	laminin, beta 3	1.21	1.36	n.s.	6
IFIT3	interferon-induced protein with tetratricopeptide repeats 3	1.20	3.13	1.94	6
CMPK2	cytidine monophosphate (UMP-CMP) kinase 2, mitochondrial	1.13	2.38	1.24	6
SELE	selectin E	1.11	1.26	n.s.	6
ICAM1	intercellular adhesion molecule 1	1.11	1.95	0.84	6
TNFAIP2	tumor necrosis factor, alpha-induced protein 2	1.08	1.67	n.s.	6
IRF1	interferon regulatory factor 1	1.02	1.55	n.s.	6
IL1A	interleukin 1, alpha	1.02	2.66	1.65	6
MRGPRX3	MAS-related GPR, member X3	1.02	1.60	0.58	6
ZC3H12A	zinc finger CCCH-type containing 12A	1.01	1.88	0.87	6
PLAT	plasminogen activator, tissue	1.01	1.27	n.s.	6
MMP10	matrix metallopeptidase 10 (stromelysin 2)	2.04	2.25	n.s.	12
MMP1	matrix metallopeptidase 1 (interstitial collagenase)	1.79	1.39	n.s.	12
STC1	stanniocalcin 1	1.50	1.87	n.s.	12
ANPEP	alanyl (membrane) aminopeptidase	1.35	1.04	n.s.	12
SERPINA3	serpin peptidase inhibitor, clade A (alpha-1 antiproteinase, antitrypsin), member 3	1.29	2.66	1.37	12
LCN2	lipocalin 2	1.08	2.31	1.24	36

The relative fold change for these genes when comparing HMPV and HMPVΔSH infections with mock infected cells is provided in column 4 and 5.

* not significantly differentially regulated.

**Table 3:** Down-regulated genes (FDR < =  0.05) with a minimum fold change of 1 (2log values) in A549 cells infected with HMPV compared with HMPVΔSH infected cells (column 3).

symbol	Gene name	HMPV vs HMPVΔSH	HMPV vs mock	HMPVΔSH vs mock	hours p.i.
IL28A	interleukin 28A (interferon, lambda 2)	–1.762	2.633	4.395	12
IDO1	indoleamine 2,3-dioxygenase 1	–1.65	1.374	3.024	12
NA	NA	–1.308	–1.658	n.s.*	12
TNFSF10	tumor necrosis factor (ligand) superfamily, member 10	–1.305	2.125	3.43	12
IL29	interleukin 29 (interferon, lambda 1)	–1.3	1.804	3.104	12
LAMP3	lysosomal-associated membrane protein 3	–1.219	2.007	3.226	12
IFNB1	interferon, beta 1, fibroblast	–1.188	2.29	3.478	12
PKDCC	protein kinase domain containing, cytoplasmic homolog	–1.109	n.s.	1.098	12
NA	NA	–1.1	–1.049	n.s.	12
RARRES3	retinoic acid receptor responder (tazarotene induced) 3	–1.1	0.866	1.966	12
TNFSF13B	tumor necrosis factor (ligand) superfamily, member 13b	–1.057	2.072	3.129	12
NA	NA	–1.04	–0.523	0.517	12
APOL6	apolipoprotein L, 6	–1.653	n.s.	2.114	36

The relative fold change for these genes when comparing HMPV and the HMPVΔSH infections with mock infected cells is provided in column 4 and 5.

* not significantly differentially regulated.

Differences in host gene expression between HMPV- and HMPVΔSH-infected cells were almost exclusively (33 out of 35 genes) observed at early timepoints (6 and 12 hours p.i.), and differences in gene expression levels were relatively low (up to 3, 2log values). Moreover, the upregulation or downregulation of these genes is similar for both HMPV- and HMPVΔSH-infected cells compared to mock-infected cells. Only due to small differences in timing of these responses, these genes were found to be differentially expressed. Taken together, HMPV infection induced a profound perturbation of mRNA expression levels, but the mRNA expression profiles of HMPV and HMPVΔSH were remarkably similar if not identical, thus the expression of the HMPV SH protein during infection does not have an effect on host gene expression.

### Peptide profiling of HMPV and HMPVΔSH infected A549 cells

Samples of A549 cells inoculated with HMPV and HMPVΔSH that were used for the microarray analysis were also analyzed using Matrix-assisted laser desorption/ionisation (MALDI) Fourier Transform Ion Cyclotron Resonance Mass Spectrometer (FT-MS) and LC MALDI Time-of-Flight (TOF)/TOF. For peptide profiling, samples collected at 12, 24 and 36 hours p.i. were analyzed. When applying a signal to noise ratio of 10, approximately 800 mass peaks could be identified in a typical A549 derived peptide profile. On average, these peaks relate to 500 peptides originating from about 400 distinct proteins. When comparing peptide profiles of HMPV infected cells with mock infected cells obtained at the same time point, up to 100 mass peaks differing in signal intensity were observed. In addition to host peptides that were differentially expressed upon infection, several peptides specific for the HMPV N, P and M2.1 proteins were detected at 12, 24 and 36 p.i. in the HMPV and HMPVΔSH infected cells but not in the mock infected cells (data not shown). The HMPV SH protein was not detected, which could be due to low expression levels, the relatively small size of the protein or glycosylation status. When comparing the peptide profiles derived from HMPV and HMPVΔSH inoculated cells directly, not a single host derived peptide was differentially expressed. Thus, no effect of the HMPV SH protein on protein expression profiles in infected A549 cells was detected.

## Discussion

Despite years of research, little is known about the function of the SH protein of members of the subfamily *Pneumovirinae*. For HRSV the SH protein can be deleted with minimal effect on virus replication *in vitro* [12], [17], [28]. HMPV SH deletion mutants replicated as efficient as wild type HMPV in Vero-118 cells and did not display differences in CPE and plaque size. This is in agreement with the results described for HMPV by others using LLC-MK2 cells [12]. Replication curves of HMPV and HMPVΔSH in cell lines such as MDBK, 293T and A549 cells also did not reveal any differences in replication kinetics or CPE (data not shown). HMPV is known to replicate primarily in ciliated respiratory epithelial cells in macaques [26]. For that reason HPBEC might represent a more suitable *in vitro* cell culture model. HPBEC cultures consist of polarized cells with a pseudostratified mucociliary histology that is very similar to bronchial epithelium *in vivo*. HPBEC cultures are derived from lung tissue samples and contain a wide variety of cells, including mucus producing cells and ciliated cells. Similar to RSV [29], both HMPV and HMPVΔSH were primarily detected in ciliated cells. No differences were observed in cell-to-cell spread, the number of virus infected cells or CPE in these cell cultures.

The deletion of the SH protein of PIV5 resulted in an increase of CPE and apoptosis in several cell lines, such as A549, MDBK, HeLaT4 and L929 [24]. The PIV5 SH protein was shown to block the TNF-α-mediated apoptosis in L929 cells. Assays with chimeric PIV5ΔSH expressing the SH proteins of other viruses revealed that the MuV and RSV SH proteins have a similar function in blocking the TNF-α-induced NF-κB activation [21], [22]. Infection of Vero-118 with PIV5ΔSH resulted in a major increase in CPE compared to PIV5 (data not shown). In contrast Vero-118 or A549 cells infected with HMPV and HMPVΔSH did not display this phenotype. Thus, the deletion of HMPV SH does not result in a similar change of phenotype as the deletion of PIV5 SH *in vitro*.

RNA microarray analysis of A549 cells infected with HMPV or HMPVΔSH revealed that more than 500 genes were differentially expressed over time (cumulative count). Similar results have been obtained by Bao *et al* [30]. HMPV infection altered the transcriptome network considerably but direct comparison of HMPV and HMPVΔSH infections revealed only marginal differences between the genome wide expression profiles. Furthermore, the differences were restricted to the first 12 hours of infection and mainly involved innate immunity genes that are induced by both HMPV and HMPVΔSH infections when compared to mock-infected cells. Since the early and transient gene expression differences are not followed by gene expression differences at later time points when the expression level of many genes change, we concluded that the SH protein is transcriptionally inert and does not affect the *in vitro* host response phenotype. Other groups have described reduced interleukin 6 (IL-6) and IL-8 expression in A549 cells infected with HMPV compared to HMPVΔSH, although this difference was only found at 3 and 6 h p.i., and disappeared at 15 h p.i. [14]. It was hypothesised that the down regulation of IL-6 and IL-8 was a result of the inhibition of NF-κB dependent transcription by the SH protein, since NF-κB is required for IL-8 and IL-6 expression, thereby suggesting a possible role of the SH protein in blocking of apoptosis [31], [32]. However, microarray analysis of HMPV and HMPVΔSH infected A549 infected cells did not reveal differential expression of genes encoding IL-6 and IL-8 or other genes involved in the apoptosis pathways, thereby refuting a possible role of HMPV SH in TNF-α-induced NF-κB activation.

Expression levels of mRNA are not always consistent with protein levels and post-translational protein modifications may be altered in virus infected cells [33]. Therefore, protein expression profiles of HMPV and HMPVΔSH infected A549 cells were generated. Approximately 100 peptides were found to be differentially expressed between HMPV and mock infected cells, some of which represented viral proteins. However, there were no differences in protein expression profiles between HMPV and HMPVΔSH infected A549 cells.

From these results, we conclude that the function of SH is still not clear. Since HMPV displays a high mutation rate and because SH is present in all primary virus isolates, it seems unlikely that SH has no function *in vivo*. Frequent frameshift and point mutations in the SH gene have been observed in *in vitro* studies by some groups [34]. This phenomenon may be cell type specific, since repeated passage of HMPV-B1 viruses in Vero-118 cells did not result in mutations in the SH ORF [35]. This is suggestive of lack of SH function in *in vitro* models. HMPV SH deletion mutants were not attenuated in hamsters and only slightly attenuated in chimpanzees compared to wild type HMPV [12], [14], [18]. Inoculation of mice with HMPV SH deletion mutants resulted in enhanced secretion of proinflammatory mediators compared with wild type HMPV, although both viruses replicated to similar titers [14]. The lack of a clear phenotype could be due to the fact that the animal models used do not mimic the human physiology sufficiently to read out a possible phenotype for the HMPV SH deletion mutants. Therefore, future studies on SH function of metapneumoviruses should focus on the natural hosts, birds and humans for AMPV and HMPV respectively. From the studies conducted so far, we conclude that the function of the HMPV SH protein is yet to be discovered.

## Methods

### Cells and media

Vero-118 cells [26] were cultured in Iscove’s Modified Dulbecco’s medium (IMDM, BioWhittaker, Verviers, Belgium) supplemented with 10 % Fetal Calf serum (FCS), 100 IU of penicillin ml^-1^, 100 µg of streptomycin ml^-1^, and 2 mM glutamine as described previously [7]. 293T cells were grown in Dulbecco’s modified eagle medium (DMEM, BioWhittaker, Verviers, Belgium), supplemented with 10% FCS, nonessential amino acids, 100 IU of penicillin/ml, 100 µg of streptomycin/ml and 2 mM glutamine. Baby hamster kidney cells stably expressing T7 RNA polymerase (BSR-T7), a kind gift of Dr K. Conzelmann, [36] were grown in DMEM supplemented with 10 % FCS, nonessential amino acids, 100 IU of Penicillin ml^-1^, 100 µg of streptomycin ml^-1^, 2 mM glutamine and 0.5 mg ml^-1^ of G418 (Invitrogen, Breda, The Netherlands). For HMPV rescue, Vero-118 cells and BSR-T7 cells were co-cultured in DMEM supplemented with 3 % FCS, 100 IU of penicillin ml^-1^, 100 µg of streptomycin ml^-1^, 2 mM glutamine, and 0.25 mg trypsin ml^-1^. For virus propagation and titration, Vero-118 cells were grown in IMDM supplemented with 4 % bovine serum albumin fraction V (Invitrogen, Breda), 100 IU of penicillin, 2 mM glutamine, and 3.75 µg of trypsin ml^-1^. A549 cells were cultured in HAM F12 medium containing 100 IU of penicillin ml^-1^, 100 µg of streptomycin ml^-1^, 2 mM glutamine and 10 % FCS (Hyclone, Logan, USA). For virus infection, A549 were grown in HAM F12 medium containing 100 IU of penicillin ml^-1^, 100 µg of streptomycin ml^-1^, 2 mM glutamine and 4 % bovine serum albumin fraction V (Invitrogen).

Subcultures of HPBEC were obtained from resected lung tissue as previously described [27]. Briefly, HPBEC were grown in keratinocyte serum-free medium (KSFM, Invitrogen) supplemented with 0.2 ng/ml epithelial growth factor (Invitrogen), 25 µg/ml bovine pituitary extract (Invitrogen), 1 µM isoproterenol, 20 U/ml penicillin and 20 µg/ml streptomycin. For air-liquid inferphase cultures, cells were seeded (1.5×10^5 ^cells/cm^2^) in DMEM/ bronchial epithelial cell medium (BEGM) 1 1 medium with supplements (Bulletkit; Lonza, Breda, The Netherlands) on 24-well transwell inserts (0.4 µm pore-size, 6 mm diameter; Corning Incorporated, Schiphol-Rijk, The Netherlands) that were precoated with 10 µg/ml bovine fibronectin (Sigma, Zwijndrecht, The Netherlands), 30 µg/ml Purecol (Inamed, Fremont, CA), and 10 µg/ml bovine serum albumin in PBS. When cell layers were confluent the apical medium was removed and basal medium was replaced with DMEM/BEGM medium supplemented with 10 nM retinoic acid (Sigma, Zwijndrecht, The Netherlands). Cells were allowed to differentiate for 2 weeks.

Differentiated air-liquid interphase cultures were pretreated with LPC prior to inoculation with HMPV. Basal medium of air-liquid interphase cultures was replaced with HBS (150 mM NaCl, 20 mM HEPES pH 7.5, 1 mM MgCl_2_ and 1 mM CaCl_2_) and the apical side washed 3x with HBS with 75 ug/ml LPC for 10 min. Basal HBS was then replaced with DMEM/BEGM medium and HMPV was added. Six days after inoculation a double staining was performed, infected cells were visualized by immunostaining with HMPV specific polyclonal anti-serum and ciliated cells were detected by staining with anti ß-tubulin antibodies.

### Full-length cDNA vectors

The full-length cDNA plasmids for HMPV-B1 (NL/1/99) and HMPV-B1 expressing the green fluorescent protein (HMPV-GFP) have been described previously [7], [25]. For the construction of the full-length HMPV-B1 cDNA plasmids lacking the SH ORF, fragments of HMPV-B1 spanning the gene start (GS) of SH or the gene end (GE) of SH were amplified by PCR by primers flanked by type II restriction sites, and cloned into pBluescript SK^+^. Using the type II restriction sites, fragments were cloned such that the GS of SH, was directly ligated to the GE of SH, thus omitting the SH ORF. Using unique restriction sites, the fragment lacking the SH ORF was swapped back into the full-length HMPV-B1 cDNA plasmids resulting in HMPVΔSH and HMPVΔSH-GFP. All plasmids were sequenced to ensure the absence of undesired mutations. All primer sequences are available upon request.

### Recovery of recombinant virus

Recombinant HMPV was generated as described previously [7]. Briefly, BSR-T7 cells were co-transfected for 5 hours with 5 µg of the full-length HMPV cDNA plasmid, 2 µg pCITE-N, 2 µg pCITE-P, 1 µg pCITE-L and 1 µg pCITE-M2.1 using Lipofectamine 2000 (Invitrogen). After transfection, the media was replaced with fresh media supplemented with trypsin. Three days after transfection, the BSR-T7 cells were scraped and cocultured with Vero-118 cells for 8 days. After one freeze–thaw cycle, cell-free supernatants were purified and concentrated using a 20–60% (w/w) sucrose gradient. Viral RNA was isolated after recovery of the virus and sequenced to ensure no point mutations or frame shifts had occurred in the SH ORF.

### Virus titrations

Viruses were propagated in Vero-118 cells and virus titers were determined as described previously [7]. Briefly, confluent monolayers of Vero-118 cells in 96-well plates were spin-inoculated (15 min., 2000 x *g*) with 100 µl of ten fold serial dilutions of each sample and incubated at 37°C. After 2 hours and again after 3-4 days, the inoculum was replaced with fresh infection media. Seven days after inoculation, infected wells were identified by immunofluorescence assays with HMPV-specific polyclonal antiserum raised in guinea pigs, as described previously [1]. Titers expressed as 50 % tissue culture infectious dose (TCID_50_) were calculated as described by Reed and Muench [37].

### Replication curves

Replication curves were generated as described previously [7]. Twenty-five cm^2^ flasks containing confluent Vero-118 cells were inoculated for 2 h at 37°C with HMPV, HMPV-GFP, HMPVΔSH or HMPVΔSH-GFP at a multiplicity of infection (MOI) of 0.1. After adsorption of the virus to the cells, the inoculum was removed and cells were washed two times with medium before addition of 7 ml of fresh medium and incubation at 37°C. Every day, 0.5 ml of supernatant was collected and replaced by fresh media. Plaque assays were performed to determine viral titers [7].

### Protein electrophoresis and Western blot assay

239T cells were transfected with pCAGGS or pCAGGS expressing the HMPV-B1 SH protein (pCAGGS-SH, a kind gift of Dr L. Martínez-Sobrido) using the CaPO_4_ precipitation method and harvested 48 h after transfection [38]. Vero-118 cells were infected with HMPV or HMPVΔSH and cells were subsequently harvested 7 days after infection, followed by virus purification on a 60-20% sucrose gradient (2 h, 27000 rpm). All samples were lysed in hot lysis buffer (1% sodium dodecyl sulfate (SDS]), 100 mM NaCl, 10 mM EDTA, 10 mM Tris-HCl at pH 7.5), treated with 3X dissociation loading buffer (2% SDS, 0.01 dithiothreitol, 0.02 M Tris-HCl at pH 6.8) for 5 min at 96°C, and analyzed on a 12.5 % SDS-polyacrylamide gels. Proteins were electrotransferred onto nitrocellulose membranes. The blots were incubated overnight at 4°C in blocking buffer (PBS with 5% nonfat dried milk and 0.05% Tween) and subsequently incubated with a 1 200 dilution of rabbit antisera (rabbit anti-SH NL/1/99 raised against a mixture of peptides representing aa 2 to 16 and 95 to 110 of SH) in blocking buffer for 2 h at room temperature. Blots were washed with PBS containing 0.05% Tween and incubated for 1 h with swine anti-rabbit horseradish peroxidase (Dako, Denmark) at a dilution of 1 3000 in blocking buffer, washed again, and developed with ECL Western blotting detection reagents (GE Healthcare, United Kingdom).

### Orbitrap MS analysis

After addition of 80% ethanol/water at -20°C the samples were incubated at room temperature for one hour. Next, the samples were centrifuged at 10000 g at 4°C and pellets were sonified in 50 µl 0.1% Rapigest for 1 min using the Ultrasonic Disruptor Sonifier (Brandson Ultrasonic, Danbury, CT) at 70% amplitude at a maximum temperature of 25°C. The samples were subsequently reduced and alkylated with 1,4-dithiothreitol and iodoacetamide and digested overnight using trypsin (Promega, Madison, WI). The digestion was terminated by adding trifluoracetic acid (TFA, Biosolve Valkenswaard) to a final concentration of 0.5% (pH<2). After incubation for 45 min at 37°C the samples were centrifuged at 14000 g for 40 min and analyzed using Orbitrap MS. Peptide measurements were carried out on an Ultimate 3000 nano LC system (Dionex, Germering, Germany) on-line coupled to a hybrid linear ion trap/Orbitrap MS (LTQ Orbitrap XL; Thermo Fisher Scientific, Germany). Digests were loaded onto a C18 trap column (C18 PepMap, 300 µm ID ×5 mm, 5 µm particle size, 100 Å pore size; Thermo Fisher Scientific, Amsterdam, The Netherlands) and desalted for 10 minutes using a flow rate of 20 µL/min 0.1% TFA. Next, the trap column was switched on-line with the analytical column (PepMap C18, 75 µm ID ×500 mm, 2 µm particle and 100 Å pore size; Thermo Fisher Scientific) and peptides were eluted with following binary gradient: 3%–25% solvent B in 120 min and 25%–50% solvent B in further 60 minutes, where solvent A consist of 0.1% formic acid in water and solvent B consists of 80% acetonitrile (ACN) and 0.08% formic acid in water. Column flow rate was set to 250 nL/min. For MS detection a data-independent acquisition method with a parent mass list of the theoretical tryptic peptides (carbamidomethylated cysteine and two miscleavages allowed) of SH protein (Table S1) was used. A high resolution survey scan recording a window between 400 and 1800 mass-to-charge (*m/z*) was performed in the Orbitrap (value of target of automatic gain control AGC 10^6^, resolution 30,000 at 400 m/z; lock mass was set to 445.120025 u (protonated (Si(CH_3_)_2_O)_6_)) [39]. Only masses that corresponded to the parent mass list within a range of 20 ppm were selected for MS/MS analysis. MS/MS spectra were searched against the virus subset of the NCBInr database (version November 5^th^, 2011, 881,102 virus sequence entries) using Mascot version 2.3 (Matrix Science, London, U.K.). Search parameters were specified as follows: (i) taxonomy, virus; (ii) enzyme, trypsin; (iii) fixed modification, carbamidomethylation of cysteine; (iv) variable modification, oxidation of methionine. We used a peptide tolerance of 10 parts per million (ppm) and a fragment tolerance 0.5 Da.

### Biological materials for RNA isolation and protein purification

A549 cells were seeded at 500.000 cells/well in a 6 wells plate the day before inoculation. These plates were spin-inoculated (15 min., 2000 X *g*) with HMPV or HMPVΔSH and incubated at 37°C. After 2 hours, the inoculum was replaced with fresh infection media. After 24 hours, cells were stained with HMPV-specific polyclonal antiserum and analyzed by FACS. For RNA isolation cells were washed twice with PBS and homogenized in 1 ml TRIzol^®^ reagent (Invitrogen) and stored at -80°C.

### RNA isolation, labeling, Affymetrix microarray hybridization and data analysis

The TRIzol® homogenates were processed according to the manufacturer’s instructions (Invitrogen). Total RNA was isolated and purified using the RNeasy Mini kit (Qiagen, Hilden, Germany): 250 µl of ethanol was added to the upper aqueous phase of the processed TRIzol samples and directly transferred to the RNeasy spin columns for purification. RNA concentrations and OD 260/280 ratios were measured with the NanoDrop® ND-1000 UV-VIS spectrophotometer (NanoDrop Technologies, Wilmington, USA). Assessment of total RNA quality and purity was performed with the RNA 6000 Nano assay on the Agilent 2100 bioanalyzer (Agilent Technologies, Palo Alto, CA, USA). CDNA was synthesized from total RNA using the One-Cycle Target Labeling kit (Affymetrix, Santa Clara, CA, USA). Subsequent biotin-labelled cRNA synthesis, purification and fragmentation were performed according to the manufacturer’s recommendations. Fragmented biotinylated cRNA was subsequently hybridised onto Affymetrix Human Genome U133 Plus 2.0 microarray chips. Image analysis was performed using GeneChip Operating Software. Microarray Suite software (Affymetrix) was used to generate .dat and .cel files. All processing of data and statistics were performed in Bioconductor version 2.6, run in R version 2.11.1. The raw intensity values obtained from the scanner (the CEL files) were preprocessed using the expresso function of the affy package (version 1.26.1) [40]. In the expresso function call, parameters were set to use the perfect match (PM) probe intensities only. Batchwise background correction and normalization were performed by the variance stabilization and calibration (VSN) algorithm (package version 3.16.0, ref 21). The transformed probe values were summarized into one value per probe set by the median polish method that is part of the robust multiarray averaging (RMA) method using probe set definitions provided by Brainarray (http://brainarray.mbni.med.umich.edu/Brainarray/default.asp) as defined in custom CDF package version 13.0.0. Probe set wise comparisons between the experimental conditions were performed by linear models of microarray data (Limma) (version 3.4.5) [41]. Correction for multiple testing was achieved by requiring a false discovery rate (FDR) of 0.05, calculated with the Benjamini-Hochberg procedure [42]. Pathway analysis was performed using the Interactive Pathway Analysis (IPA) module from Ingenuity Systems Inc (Redwood City, CA, USA). Microarray data are available in the ArrayExpress database (www.ebi.ac.uk/arrayexpress) under accession number E-MTAB-1152.

### Protein purification

Protein samples were processed as follows. After centrifugation of the TRIzol^®^ homogenates and removal of the RNA containing aqueous phase, proteins were isolated from the organic phase. The, organic phase was washed with acetone (-20°C) and precipitated by centrifugation at 10.000 x g three times. Next, 100 µl 0.1% w/v RapiGest^TM^ SF reagent (Walters, Milford, MA, USA), dissolved in 50 mM ammonium bicarbonate, pH 7.0 was added and sonicated with an Ultrasonic Disruptor Sonifier^®^ II (Branson Ultrasonics, Danbury, Connecticut, USA). Gold grade trypsin (Promega, Madison, WI, USA) was added to the protein solution at 0.1 g/L and incubated overnight at 37°C. Next 10 µL of 500 mM HCl was added (final concentration: 30–50 mM HCl, pH<2) and incubated for 45 min at 37°C.

Samples were then processed for proteomics-analyses, performed by a combination of two MS techniques. For peptide quantification MALDI-FT-MS was used, which measures peptide masses with an accuracy better than 1 ppm, with a dynamic range of signal intensities of circa 3–4 orders of magnitude. Identification of the peptides was performed by off-line LC-MALDI-TOF/TOF. Next, the masses of the identified peptides were linked to the MALDI-FT-MS peptide signal intensities to enable fold change analyses of identified proteins [43], as outlined below.

### MALDI-FT-MS

MALDI-FT-MS was performed as described previously [44]. Samples were spotted onto a 600/384 AnchorChip target plate (Bruker Daltonics, Leipzig, Germany) in duplicate. The MALDI-FTMS measurements were performed on a Bruker Apex Q instrument with 9.4-tesla magnet (Bruker Daltonics). For each measurement, 200 scans of 10 shots each were accumulated with 60% laser power. Mass spectra were acquired in the mass range of 800–4000 Da. FTMS spectra were processed with a Gaussian filter and two zero fillings. A standard peptide calibration mixture (Bruker Daltonics, Leipzig, Germany) was used for external calibration. To obtain better mass accuracies, an additional post-acquisition internal calibration step in DataAnalysis version 3.4, build 169 software (Bruker Daltonics) was performed. Ubiquitous actin peptide masses (1198.70545, 1515.74913, 1790.89186, 2215.06990, and 3183.61423 *m/z*) were used for internal calibration. Peak intensities were retrieved using a peak finding algorithm that determines the highest peak intensity within a 3 ppm window at both sides of each *m/z* value present above a signal to noise ratio of >4, as described previously [45].

### MALDI-TOF/TOF-MS

Trypsin digested samples were run on an Ultimate 3000^TM^ Nano LC System (Dionex, Sunnyvale, California, USA). One µl of the sample was loaded onto the monolith trap column (200 µm inner diameter x 5 cm) with a gradient of eluent A (0.05% TFA in water) and eluent B (80% ACN, 0.04% TFA in water): 0–5 min, 10% eluent B; 70 min, 50%; 71 min, 90%; 76 min, 90%; 77–100 min, 0% using a flow rate of 1.5 µl/min. Fifteen-second fractions of the sample were spotted automatically onto a 384 prespotted AnchorChip plate (Bruker Daltonics) containing α-cyano-4-hydroxycinnamic acid matrix using a robotic system (Probot Micro Fraction Collector, Dionex). To each fraction 1 µl of water was added. Finally, we used a 10 mM (NH)_4_H_2_PO_4_ in 0.1% TFA, water solution to wash the prespotted plate to remove salts. The plates were subsequently measured by automated MALDI-TOF/TOF (Ultraflex, Bruker Daltonik GmbH, Leipzig, Germany) using WARP-LC software. A file containing the MS and the MS/MS peak lists was submitted to the MASCOT search engine, version 2.1. (Matrix Science, London, UK) using the MSDB database (release 09-08-2006) allowing 150 ppm parent mass tolerance, 0.5 Dalton fragment tolerance, and one missed trypsin cleavage site.

## Supporting Information

Table S1 **Theoretical peptides based on trypsine digestion of the HMPV SH protein.**
(DOCX)

## References

[1] van den Hoogen BG, de Jong JC, Groen J, Kuiken T, de Groot R, et al (2001) A newly discovered human pneumovirus isolated from young children with respiratory tract disease. Nat Med 7: 719–724. 11385510 10.1038/89098 PMC7095854

[2] van den Hoogen BG, Doornum GJJv, Fockens JC, Cornelissen JJ, Beyer WEP, et al. (2003) Prevalence and clinical symptoms of human metapneumovirus infection in hospitalized patients. J Infect Dis 188.. 10.1086/379200 14624384

[3] Williams JV, Harris PA, Tollefson SJ, Halburnt-Rush LL, Pingsterhaus JM, et al (2004) Human metapneumovirus and lower respiratory tract disease in otherwise healthy infants and children. N Engl J Med 350: 443–450. 14749452 10.1056/NEJMoa025472 PMC1831873

[4] Kahn JS (2006) Epidemiology of human metapneumovirus. Clin Microbiol Rev 19: 546–557. 16847085 10.1128/CMR.00014-06 PMC1539100

[5] Schildgen V, van den Hoogen B, Fouchier R, Tripp RA, Alvarez R, et al (2011) Human Metapneumovirus: lessons learned over the first decade. Clin Microbiol Rev 24: 734–754. 21976607 10.1128/CMR.00015-11 PMC3194831

[6] van den Hoogen BG, Herfst S, Sprong L, Cane PA, Forleo E, et al (2004) Antigenic and genetic variability of human metapneumoviruses. Emerg Infect Dis 10: 658–666. 15200856 10.3201/eid1004.030393 PMC3323073

[7] Herfst S, de Graaf M, Schickli JH, Tang RS, Kaur J, et al (2004) Recovery of human metapneumovirus genetic lineages a and B from cloned cDNA. J Virol 78: 8264–8270. 15254198 10.1128/JVI.78.15.8264-8270.2004 PMC446134

[8] Biacchesi S, Skiadopoulos MH, Tran KC, Murphy BR, Collins PL, et al (2004) Recovery of human metapneumovirus from cDNA: optimization of growth in vitro and expression of additional genes. Virology 321: 247–259. 15051385 10.1016/j.virol.2003.12.020

[9] van den Hoogen BG, Bestebroer TM, Osterhaus AD, Fouchier RA (2002) Analysis of the genomic sequence of a human metapneumovirus. Virology 295: 119–132. 12033771 10.1006/viro.2001.1355

[10] Fearns R, Collins PL (1999) Role of the M2-1 transcription antitermination protein of respiratory syncytial virus in sequential transcription. J Virol 73: 5852–5864. 10364337 10.1128/jvi.73.7.5852-5864.1999 PMC112646

[11] Buchholz UJ, Biacchesi S, Pham QN, Tran KC, Yang L, et al (2005) Deletion of M2 gene open reading frames 1 and 2 of human metapneumovirus: effects on RNA synthesis, attenuation, and immunogenicity. J Virol 79: 6588–6597. 15890897 10.1128/JVI.79.11.6588-6597.2005 PMC1112115

[12] Biacchesi S, Skiadopoulos MH, Yang L, Lamirande EW, Tran KC, et al (2004) Recombinant human Metapneumovirus lacking the small hydrophobic SH and/or attachment G glycoprotein: deletion of G yields a promising vaccine candidate. J Virol 78: 12877–12887. 15542640 10.1128/JVI.78.23.12877-12887.2004 PMC525014

[13] Teng MN, Whitehead SS, Collins PL (2001) Contribution of the respiratory syncytial virus G glycoprotein and its secreted and membrane-bound forms to virus replication in vitro and in vivo. Virology 289: 283–296. 11689051 10.1006/viro.2001.1138

[14] Bao X, Kolli D, Liu T, Shan Y, Garofalo RP, et al (2008) Human metapneumovirus small hydrophobic protein inhibits NF-kappaB transcriptional activity. J Virol 82: 8224–8229. 18550666 10.1128/JVI.02584-07 PMC2519579

[15] Yunus AS, Govindarajan D, Huang Z, Samal SK (2003) Deduced amino acid sequence of the small hydrophobic protein of US avian pneumovirus has greater identity with that of human metapneumovirus than those of non-US avian pneumoviruses. Virus Res 93: 91–97. 12727346 10.1016/s0168-1702(03)00074-1

[16] Whitehead SS, Bukreyev A, Teng MN, Firestone CY, St Claire M, et al (1999) Recombinant respiratory syncytial virus bearing a deletion of either the NS2 or SH gene is attenuated in chimpanzees. J Virol 73: 3438–3442. 10074199 10.1128/jvi.73.4.3438-3442.1999 PMC104109

[17] Bukreyev A, Whitehead SS, Murphy BR, Collins PL (1997) Recombinant respiratory syncytial virus from which the entire SH gene has been deleted grows efficiently in cell culture and exhibits site-specific attenuation in the respiratory tract of the mouse. J Virol 71: 8973–8982. 9371553 10.1128/jvi.71.12.8973-8982.1997 PMC230197

[18] Biacchesi S, Pham QN, Skiadopoulos MH, Murphy BR, Collins PL, et al (2005) Infection of nonhuman primates with recombinant human metapneumovirus lacking the SH, G, or M2-2 protein categorizes each as a nonessential accessory protein and identifies vaccine candidates. J Virol 79: 12608–12613. 16160190 10.1128/JVI.79.19.12608-12613.2005 PMC1211552

[19] Perez M, Garcia-Barreno B, Melero JA, Carrasco L, Guinea R (1997) Membrane permeability changes induced in Escherichia coli by the SH protein of human respiratory syncytial virus. Virology 235: 342–351. 9281514 10.1006/viro.1997.8696

[20] Gan SW, Tan E, Lin X, Yu D, Wang J, et al.. (2012) The small hydrophobic protein of the human respiratory syncytial virus forms pentameric ion channels. J Biol Chem. 10.1074/jbc.M111.332791 PMC3397895 22621926

[21] Wilson RL, Fuentes SM, Wang P, Taddeo EC, Klatt A, et al (2006) Function of small hydrophobic proteins of paramyxovirus. J Virol 80: 1700–1709. 16439527 10.1128/JVI.80.4.1700-1709.2006 PMC1367141

[22] Fuentes S, Tran KC, Luthra P, Teng MN, He B (2007) Function of the respiratory syncytial virus small hydrophobic protein. J Virol 81: 8361–8366. 17494063 10.1128/JVI.02717-06 PMC1951288

[23] Lin Y, Bright AC, Rothermel TA, He B (2003) Induction of apoptosis by paramyxovirus simian virus 5 lacking a small hydrophobic gene. J Virol 77: 3371–3383. 12610112 10.1128/JVI.77.6.3371-3383.2003 PMC149502

[24] He B, Lin GY, Durbin JE, Durbin RK, Lamb RA (2001) The SH integral membrane protein of the paramyxovirus simian virus 5 is required to block apoptosis in MDBK cells. J Virol 75: 4068–4079. 11287556 10.1128/JVI.75.9.4068-4079.2001 PMC114152

[25] de Graaf M, Herfst S, Schrauwen EJ, van den Hoogen BG, Osterhaus AD, et al (2007) An improved plaque reduction virus neutralization assay for human metapneumovirus. J Virol Methods 143: 169–174. 17420056 10.1016/j.jviromet.2007.03.005

[26] Kuiken T, Van Den Hoogen BG, Van Riel DA, Laman JD, Van Amerongen G, et al (2004) Experimental Human Metapneumovirus Infection of Cynomolgus Macaques (Macaca fascicularis) Results in Virus Replication in Ciliated Epithelial Cells and Pneumocytes with Associated Lesions throughout the Respiratory Tract. Am J Pathol 164: 1893–1900. 15161626 10.1016/S0002-9440(10)63750-9 PMC1615765

[27] van Wetering S, Zuyderduyn S, Ninaber DK, van Sterkenburg MA, Rabe KF, et al (2007) Epithelial differentiation is a determinant in the production of eotaxin-2 and -3 by bronchial epithelial cells in response to IL-4 and IL-13. Mol Immunol 44: 803–811. 16740309 10.1016/j.molimm.2006.04.008

[28] Jin H, Zhou H, Cheng X, Tang R, Munoz M, et al (2000) Recombinant respiratory syncytial viruses with deletions in the NS1, NS2, SH, and M2-2 genes are attenuated in vitro and in vivo. Virology 273: 210–218. 10891423 10.1006/viro.2000.0393

[29] Zhang L, Peeples ME, Boucher RC, Collins PL, Pickles RJ (2002) Respiratory syncytial virus infection of human airway epithelial cells is polarized, specific to ciliated cells, and without obvious cytopathology. J Virol 76: 5654–5666. 11991994 10.1128/JVI.76.11.5654-5666.2002 PMC137037

[30] Bao X, Sinha M, Liu T, Hong C, Luxon BA, et al (2008) Identification of human metapneumovirus-induced gene networks in airway epithelial cells by microarray analysis. Virology 374: 114–127. 18234263 10.1016/j.virol.2007.12.024 PMC2777699

[31] Brasier AR, Jamaluddin M, Casola A, Duan W, Shen Q, et al (1998) A promoter recruitment mechanism for tumor necrosis factor-alpha-induced interleukin-8 transcription in type II pulmonary epithelial cells. Dependence on nuclear abundance of Rel A, NF-kappaB1, and c-Rel transcription factors. J Biol Chem 273: 3551–3561. 9452482 10.1074/jbc.273.6.3551

[32] Garofalo R, Sabry M, Jamaluddin M, Yu RK, Casola A, et al (1996) Transcriptional activation of the interleukin-8 gene by respiratory syncytial virus infection in alveolar epithelial cells: nuclear translocation of the RelA transcription factor as a mechanism producing airway mucosal inflammation. J Virol 70: 8773–8781. 8971006 10.1128/jvi.70.12.8773-8781.1996 PMC190974

[33] Skiba M, Mettenleiter TC, Karger A (2008) Quantitative whole-cell proteome analysis of pseudorabies virus-infected cells. J Virol 82: 9689–9699. 18653448 10.1128/JVI.00995-08 PMC2546971

[34] Biacchesi S, Murphy BR, Collins PL, Buchholz UJ (2007) Frequent frameshift and point mutations in the SH gene of human metapneumovirus passaged in vitro. J Virol 81: 6057–6067. 17376897 10.1128/JVI.00128-07 PMC1900297

[35] Herfst S, de Graaf M, Schrauwen EJ, Sprong L, Hussain K, et al (2008) Generation of temperature-sensitive human metapneumovirus strains that provide protective immunity in hamsters. J Gen Virol 89: 1553–1562. 18559924 10.1099/vir.0.2008/002022-0

[36] Buchholz UJ, Finke S, Conzelmann KK (1999) Generation of bovine respiratory syncytial virus (BRSV) from cDNA: BRSV NS2 is not essential for virus replication in tissue culture, and the human RSV leader region acts as a functional BRSV genome promoter. J Virol 73: 251–259. 9847328 10.1128/jvi.73.1.251-259.1999 PMC103829

[37] Reed LJ, Muench H (1938) A simple method of estimating fifty percent end points. J Hyg 27: 493–497.

[38] Pear WS, Nolan GP, Scott ML, Baltimore D (1993) Production of high-titer helper-free retroviruses by transient transfection. Proc Natl Acad Sci U S A 90: 8392–8396. 7690960 10.1073/pnas.90.18.8392 PMC47362

[39] Olsen JV, de Godoy LM, Li G, Macek B, Mortensen P, et al (2005) Parts per million mass accuracy on an Orbitrap mass spectrometer via lock mass injection into a C-trap. Mol Cell Proteomics 4: 2010–2021. 16249172 10.1074/mcp.T500030-MCP200

[40] Gautier L, Cope L, Bolstad BM, Irizarry RA (2004) affy--analysis of Affymetrix GeneChip data at the probe level. Bioinformatics 20: 307–315. 14960456 10.1093/bioinformatics/btg405

[41] Smyth GK (2004) Linear models and empirical bayes methods for assessing differential expression in microarray experiments. Stat Appl Genet Mol Biol 3: Article3. 16646809 10.2202/1544-6115.1027

[42] Benjamini Y, Hochberg Y (1995) Controlling the False Discovery Rate: a Practical and Powerful Approach to Multiple Testing. J R Statist Soc B 51: 289–300.

[43] Dekker LJ, Burgers PC, Guzel C, Luider TM (2007) FTMS and TOF/TOF mass spectrometry in concert: identifying peptides with high reliability using matrix prespotted MALDI target plates. J Chromatogr B Analyt Technol Biomed Life Sci 847: 62–64. 10.1016/j.jchromb.2006.08.031 16963321

[44] Mustafa DA, Burgers PC, Dekker LJ, Charif H, Titulaer MK, et al (2007) Identification of glioma neovascularization-related proteins by using MALDI-FTMS and nano-LC fractionation to microdissected tumor vessels. Mol Cell Proteomics 6: 1147–1157. 17360931 10.1074/mcp.M600295-MCP200

[45] Titulaer MK, Siccama I, Dekker LJ, van Rijswijk AL, Heeren RM, et al (2006) A database application for pre-processing, storage and comparison of mass spectra derived from patients and controls. BMC Bioinformatics 7: 403. 16953879 10.1186/1471-2105-7-403 PMC1594579

