# Supplementary material for: Small Hydrophobic Protein of Human Metapneumovirus Does Not Affect Virus Replication and Host Gene Expression In Vitro
Source: PLoS One. 2013 Mar 6;8(3):e58572. doi: 10.1371/journal.pone.0058572 (PMC3590193; doi:10.1371/journal.pone.0058572)
Supplement: Table S1 — Theoretical peptides based on trypsine digestion of the HMPV SH protein. (DOCX) [file pone.0058572.s001.docx]

**Table S1.** Theoretical peptides based on trypsine digestion of the HMPV SH protein

| LILALLTFFTATITVNYIK |
| --- |
| AECHCLHTTEWGCLHP |
| GTDCEEPTALCDK |
| SDGSSETCNQLK |
| VENNLQACQPK |
| FLCSGFTNSK |
| DSDTCWR |
| HTNSVTK |
| TLDVIK |
| VLIALK |
| NESDK |
| TIVEK |
| LIQR |
